# Early Visual, Refractive, and Astigmatism Vector Outcomes of StreamLight Trans-PRK for High Astigmatism (≥ 2.00 D)

**DOI:** 10.22336/rjo.2026.32

**Published:** 2026

**Authors:** Silvia Victoria Prodescu, Paul Filip Curcă, Călin Petru Tătaru, Cătălina Ioana Tătaru

**Affiliations:** 1 Department of Ophthalmology, Doctoral School, “Carol Davila” University of Medicine and Pharmacy, Bucharest, Romania; 2 Department of Ophthalmology, Alcor Clinic, Bucharest, Romania; 3 Department of Ophthalmology, “Dr. Carol Davila” Central Military Emergency University Hospital, Bucharest, Romania; 4 “Carol Davila” University of Medicine and Pharmacy, Bucharest, Romania; 5 Department of Ophthalmology, “Prof. Dr. Mircea Olteanu” Clinical Institute for Ophthalmological Emergencies, Bucharest, Romania

**Keywords:** transepithelial photorefractive keratectomy, high astigmatism, vector analysis, refractive outcomes, corneal refractive surgery, WTR = With-the-rule (astigmatism), ATR = Against-the-rule (astigmatism), Trans-PRK = transepithelial photorefractive keratectomy, FS-LASIK = femtosecond laser-assisted keratomileusis, SMILE = small incision lenticule extraction, ORA = Ocular Response Analyzer, LASEK = laser-assisted sub-epithelial keratomileusis, CH = corneal hysteresis, CRF = corneal resistance factor, RWE = real-world evidence, UDVA = uncorrected distance visual acuity, CDVA = corrected distance visual acuity, ETDRS = Early Treatment of Diabetic Retinopathy Study, logMAR = logarithm of the minimum angle of resolution, BSS = balanced salt solution, TIA = Target-induced astigmatism, SIA = Surgically-induced astigmatism, DV = Difference vector, CI = Correction index, IOS = Index of success, ME = Magnitude of error, AoE = Angle of error, D = Diopter, n or N = number of, SE or SEQ = Spherical Equivalent, SD = Standard deviation, DEQ = Defocus equivalent, K1 = Flat keratometry, K2 = Steep keratometry, μm = micrometer(s)/micron(s), CET = Central epithelial thickness, RA = Refractive astigmatism

## Abstract

**Objective:**

To report the early refractive, visual, and astigmatic-vector outcomes of single-step StreamLight™ transepithelial photorefractive keratectomy (Trans-PRK) on the WaveLight EX500 platform in eyes with high preoperative astigmatism (≥ 2.00 dioptres, D).

**Methods:**

Single-centre cohort study of 171 consecutive eyes of 107 patients (mean age 27.87 ± 6.56 years; 37.4% female) treated by a single surgeon. Astigmatic outcomes were analyzed by the Alpins method at the corneal plane. A subset of 57 eyes with a mean follow-up of 10.3 ± 7.4 months underwent stability sub-analysis.

**Results:**

Mean preoperative cylinder was 3.29 ± 0.81 D and mean spherical equivalent (SE) was −2.73 ± 2.56 D. At one month, mean UDVA was 0.05 ± 0.09 logMAR, mean residual cylinder was 0.65 ± 0.44 D, and mean SE was −0.22 ± 0.47 D. SE was within ±0.50 D of intended in 71.3% of eyes; cylinder was ≤ 0.50 D in 53.2%. Mean efficacy and safety indices were 1.04 ± 0.22 and 1.10 ± 0.22, respectively, with no eye losing two or more lines of CDVA. Vector analysis yielded a correction index of 1.09 ± 0.16, an index of success of 0.21 ± 0.14, and 99% of eyes within ±15° of the intended axis. Stability sub-analysis showed minimal change in SE and an unchanged cylinder between 1 month and a mean follow-up of 10.3 ± 7.4 months (SE −0.14 to −0.07 D).

**Discussion:**

Trans-PRK delivered good early visual, refractive, and astigmatic vector outcomes. Axis precision was high with a mean angle of error of −0.81 ± 5.32°, 80% of eyes within ±5°, and 99% within ±15° of the intended axis. Interpretation of vectorial results is subject to the specificity of the 1-month post-Trans-PRK central epithelial thickness (CET decreases in the first week, returns to baseline at 1 month, and progressively increases up to 6 months).

**Conclusions:**

Single-step StreamLight™ Trans-PRK on the WaveLight EX500 platform achieves favorable early refractive, visual, and vector-analytic outcomes in eyes with high preoperative astigmatism, with corneal-plane vector parameters that compare favorably with those reported in the published Trans-PRK literature.

## Introduction

Regular astigmatism is a common, treatable refractive error caused by a stronger power in one meridian [1,2], leading to an asymmetric refraction of light rays [3]. Uncorrected astigmatism negatively affects patients’ vision-related quality of life by increasing glare and haloes, impairing night-driving visibility, and increasing the risk of falls or physical injury [3]. Prolonged vision impairment can lead to significant eyestrain and headaches for patients, increasing the difficulty of everyday activities and negatively affecting professional life [3]. Surgical correction of astigmatism offers powerful corrective potential, restoring vision quality and greatly improving the patient’s quality of life [1-3].

Classified by source, astigmatism can be corneal, lenticular, or retinal [1]. Corneal astigmatism is the most common form of astigmatism and is secondary to abnormalities in the anterior and/or posterior corneal curvature [1,4]. Curvatural lenticular astigmatism originates from abnormalities of the crystalline lens shape, such as in lenticonus [1,5]. Positional lenticular astigmatism is caused by tilting or displacement of the lens (subluxation) [1], such as encountered in ectopia lentis and/or Marfan Syndrome [6,7]. Lenticular index astigmatism is caused by progressive variations in the refractive lens index [1,8-10]. Retinal astigmatism is rare and originates from oblique placement of the macula [1], such as in retinopathy of prematurity [11] or after traditional scleral-buckling surgery of retinal detachment [12].

Classified by type, astigmatism can be regular when there is a single higher-power refractive meridian or irregular when there are several meridians of different refractive strengths and orientation in degrees [1,2]. Regular astigmatism is commonly subclassified by orientation of the higher-power meridian into with-the-rule (60-120°, WTR astigmatism) [13], against-the-rule (0-30° or 150-180°, ATR astigmatism) [13], and oblique astigmatism (between 30-60° and 120-150°) [13]. Irregular astigmatism is most commonly corneal in origin and thus often associated with the evolution of corneal conditions such as keratoconus, where the corneal shape changes from a physiological orthogonal shape to non-orthogonal with a dramatic increase in higher-order aberrations (HOAs) [14]. Irregular astigmatism cannot be fully corrected using cylindrical spectacle lenses and requires use of specialized contact-lenses such as in keratoconus management (rigid corneal, soft, hybrid or piggyback systems – rigid corneal lens fitted over a soft contact lens) [15] or refractive surgery (corneal topography-guided [16] or via toric intraocular lens implantation [17,18] which can also be combined with adjuvant corneal treatment for other significant meridians via femtosecond laser-assisted astigmatic keratotomy (FSAK) [19].

Transepithelial photorefractive keratectomy (Trans-PRK) is a technique evolved from photorefractive keratectomy (PRK) in which an excimer laser successively removes the corneal epithelium with extreme precision [20], followed by refractive stromal ablation in a single-step, no-touch method [20] that does not require creating a flap or lenticule [21] and thus offers preservation of corneal biomechanics [21-24]. As described by authors Schmack I et al., flap creation significantly alters corneal biomechanics by reducing corneal tensile strength [23]; furthermore, since flap creation requires more preoperatory stromal corneal thickness, Trans-PRK is advantageous in sparing corneal tissue, which could lead to better corneal biomechanical strength and more long-term protection from intraocular pressure (IOP) variations [25]. The impact of laser-assisted refractive surgery on corneal biomechanics is ongoing research, with authors Xin Y et al. reporting changes in four biomechanical parameters measured using the Corvis ST device (CVS, software version 1.6r2031, OCULUS Optikgeräte, Wetzlar, Germany) [26] and showing the largest reduction of corneal stiffness in femtosecond laser-assisted keratomileusis (FS-LASIK), followed by small incision lenticule extraction (SMILE), while Trans-PRK was the most preserving of corneal stiffness [26]. A large meta-analysis by Guo H et al., using the Corvis ST and Ocular Response Analyzer (ORA) devices, also reported the largest reduction in corneal biomechanical strength in FS-LASIK [27]. Continuing the analysis, Guo H et al. concluded that SMILE significantly less affected corneal stiffness than FS-LASIK [27] and further noted that PRK and laser-assisted sub-epithelial keratomileusis (LASEK) measured even better than SMILE in corneal hysteresis (CH) [27]; this difference did not present statistical significance [27] and corneal resistance factor (CRF) values even better than SMILE.

Despite the established advantages of Trans-PRK for corneal-tissue preservation and biomechanical stability, the literature evaluating its performance specifically in eyes with high preoperative astigmatism — where greater refractive load and the precision demands of accurate cylinder-axis alignment make achieving the intended outcomes more challenging — remains comparatively limited, and contemporary single-step transepithelial implementations have not been systematically characterized in this population. Considering the above-mentioned information, the use of Trans-PRK for a more minimally invasive correction of high astigmatism could be advantageous for the patient. The present study, therefore, reports the early refractive, visual, and astigmatic-vector outcomes of single-step StreamLight™ Trans-PRK on the WaveLight® EX500® platform in a cohort of consecutive eyes selected for a preoperative refractive cylinder of 2.00 dioptres or greater.

## Materials and methods

### Study design and ethics

This study included eyes with preoperative refractive astigmatism ≥ 2.00 D that underwent Trans-PRK surgery performed by a single experienced anterior segment surgeon in a private practice setting in Bucharest, Romania, between April 2021 and November 2025. The study was designed as a real-world evidence (RWE) study analyzing clinical and astigmatic vector outcomes of StreamLight™ Transepithelial photorefractive keratectomy (Trans-PRK). Data were prospectively collected and retrospectively analyzed as part of a doctoral research project conducted at “Carol Davila” University of Medicine and Pharmacy, Bucharest. The study adhered to the tenets of the Declaration of Helsinki, and all patients provided written informed consent. Ethical approval (registration number 1990/11.05.2026) was obtained from the independent ethics committee (the Local Ethics Committee for Scientific Research of the “Prof. Dr. Mircea Olteanu” Clinical Institute for Ophthalmological Emergencies, Bucharest).

### Study population

A total of 171 consecutive eyes from 107 patients (64 of whom contributed both eyes) were included. Inclusion criteria were the presence of informed consent, age ≥ 18 years, preoperative manifest refractive cylinder magnitude ≥ 2.00 dioptres (D), regardless of spherical component (myopic, hyperopic, or mixed astigmatism), and eligibility for StreamLight™ Trans-PRK procedure, as determined by clinical assessment. All cases had regular astigmatism. Of the 171 included eyes, 119 (69.6%) had myopic astigmatism, 48 (28.1%) had mixed astigmatism, and 4 (2.3%) had hyperopic astigmatism. Cases were not randomized. Exclusion criteria included withdrawal of consent for study participation, incomplete medical records, loss to follow-up before 1 month postoperatively, history of previous ocular surgery (including prior laser refractive surgery or intraocular lens implantation), previous or concomitant corneal crosslinking procedure, presence of corneal ectatic conditions such as keratoconus, presence of corneal dystrophy, cataract, visually-impacting retinal or neurodegenerative pathology, and cases planned for sequential bioptics procedures. Pre-existing amblyopia and strabismus were not applied as exclusion criteria, as these conditions are associated with high astigmatism and their inclusion reflects the real-world design of this study. The target refraction was emmetropia (0.00 D) in all cases.

### Preoperative assessment

All patients underwent a comprehensive preoperative ophthalmic evaluation, including patient history, visual acuity assessment, subjective refraction, clinical examination, ocular surface assessment, and ancillary investigations specific to refractive surgery. Preoperative data collected included uncorrected distance visual acuity (UDVA) and corrected distance visual acuity (CDVA), which were recorded using standardized digital Early Treatment of Diabetic Retinopathy Study (ETDRS) optotypes in the decimal system (maximum 1.0) and subsequently converted to Logarithm of the minimum angle of resolution (logMAR) and Snellen equivalents for analysis. For decimal-to-Snellen conversion, the lower correspondence was chosen (for example, 0.7 decimal between Snellen 20/25 and 20/32 was converted to 20/32). Manifest and cycloplegic refraction data were collected at a vertex distance of 12 mm. Keratometry, corneal topography, tomography, pachymetry, and anterior segment biometry were performed using the Alcon WaveLight® Oculyzer™ II (Wavelight AG, USA-Germany) Scheimpflug-based tomography system and the WaveLight® Topolyzer™ Vario (Wavelight AG, USA-Germany), as well as the Anterion anterior segment optical-coherence tomograph (Heidelberg Engineering GmbH, Heidelberg, Germany). Surgical planning was based on cross-referenced measurements from the multiple imaging modalities, with the surgeon and clinical team verifying agreement between keratometric, topographic, and biometric data before proceeding. Keratometric values used in the data analysis were obtained from the WaveLight® Oculyzer™ II. Soft contact lens wearers discontinued lens use 10-14 days before corneal evaluation and surgery.

### Surgical technique

All procedures were performed by a single experienced refractive surgeon (C.P. Tătaru) under topical anaesthesia. The Alcon Wavelight® Refractive Surgery System was used in all cases, comprising the WaveLight® EX500® excimer laser (500 Hz, 193 nm; Alcon Laboratories, Fort Worth, TX, USA). Integrated pupil-tracking and static cyclotorsion correction were active intraoperatively. Ablation profile calculation data was constructed via wavefront technology and provided by Wavelight® Oculyzer™ II Scheimpflug camera tomography and Wavelight® Topolyzer™ Vario. StreamLight™ Trans-PRK was performed as a single-step transepithelial ablation using the WaveLight® EX500® excimer laser. Following ablation, Mitomycin-C 0.02% was applied for 20 seconds and subsequently washed out via irrigation with balanced salt solution (BSS). A bandage soft contact lens was placed at the end of Trans-PRK procedures and removed once corneal re-epithelialization was clinically confirmed, typically by the second postoperative visit. The standard postoperative regimen at the clinic consisted of a topical antibiotic, a topical corticosteroid with gradual tapering, and artificial tears, with oral analgesia prescribed as needed. Follow-up visits were scheduled at 3 days, 10 days, and 1 month postoperatively, with additional visits planned at 3 and 6 months.

### Outcome measures, data analysis, and astigmatism vector analysis

Postoperative visual, refractive, and clinical assessment data were anonymized and collected (at the eye level as the unit of analysis) into an Excel database (Microsoft Corporation, Redmond, WA, USA). The primary endpoint of the analysis was the 1-month postoperative visit. A secondary descriptive stability sub-analysis was performed in the subgroup of eyes with at least one additional follow-up visit beyond 1 month. Statistical analyses were performed using Minitab® 20.3 (64-bit) (© Minitab, LLC, State College, PA, USA). Continuous variables are presented as mean ± standard deviation, with medians and ranges when distributions were skewed, and categorical variables as *n* (%). Visual acuity, recorded using decimal scale, was converted to logMAR for analysis, and Snellen-line equivalents were assigned for standard graph format using the lower decimal-to-Snellen correspondence (for example, decimal 0.7, between Snellen 20/25 and 20/32, was converted to 20/32). Eyes presenting preoperatively with counting-fingers vision were assigned a conventional value of logMAR 1.8 for descriptive analyses. Manifest refraction was recorded at the spectacle plane (vertex distance 12 mm), reflecting real-world clinical measurement; for astigmatism vector analysis, refractive data were converted to the corneal plane (vertex distance 0 mm). Vector analysis was performed at the corneal plane and analyzed using the Alpins method [28]. Standard terminology applies throughout as follows. Target-induced astigmatism (TIA) represents the intended astigmatic change. Surgically-induced astigmatism (SIA) represents the achieved astigmatic correction. The Difference vector (DV) represents the residual astigmatic error between the SIA and TIA vectors. The Correction index (CI) is calculated as the SIA/TIA ratio, has an ideal value of 1.0, and is used to describe under- and overcorrection as follows: values < 1.0 indicate undercorrection, and values > 1.0 indicate overcorrection. The Index of Success (IOS) is calculated as the DV/TIA ratio and has an ideal value of 0 (no residual astigmatism). Magnitude of error (ME) is defined as the arithmetic difference between SIA and TIA magnitudes. Angle of error (AoE) is defined as the angular difference between SIA and TIA axes [28]. The mEYEstro software (version 2.5) was used to generate the standardized graphical reporting format for refractive surgery outcomes [29]. For single-angle vector plots, the graphical output was provided using AstigMATIC (version 2.0) [30]. The recently released AstigMETRICS (version 1.0) [31] was used for vector summary metrics. mEYEstro [29], AstigMATIC [30], and AstigMETRICS [31] software have all been developed by Mathieu Gauvin & Avi Wallerstein, and all analysis outcomes generated by using these software tools are cited according to the respective papers published by the aforementioned authors [29-31]. To address potential inter-eye correlation in bilateral cases, a one-eye-per-patient sensitivity analysis was performed using the left eye in patients who contributed both eyes. The sensitivity analysis covered the primary visual and refractive outcomes (UDVA, CDVA, postoperative spherical equivalent, residual cylinder magnitude) and the key vector metrics (TIA, SIA, CI, DV).

### Methodological Limitations of the Study

This study had several methodological limitations that should be considered when interpreting the results.

First, the study’s single-center, real-world design reflected the experience of a single surgeon and the case mix of a single private practice. While these aspects ensured procedural consistency, they nevertheless limited the study’s external generalizability.

Second, the primary endpoint was assessed at one month postoperatively, a timepoint at which epithelial remodeling following surface ablation might still be ongoing. As such, the “early” framing of the present analysis was intentional, and a descriptive stability sub-analysis was provided for the subgroup of eyes with longer follow-up.

Third, the duration of extended follow-up was non-uniform and reflected real-world clinical attendance rather than a protocol-defined schedule, so the stability sub-analysis was presented descriptively.

Fourth, both eyes of bilateral patients were included in the primary analysis, introducing potential inter-eye correlation; a one-eye-per-patient sensitivity analysis addressed this.

## Results

### Patient Demographics and Preoperative Characteristics

A total of 171 eyes of 107 patients undergoing StreamLight™ transepithelial photorefractive keratectomy (Trans-PRK) for preoperative refractive astigmatism ≥ 2.00 D were included in the analysis. The mean patient age at surgery was 27.87 ± 6.56 years, and 64 patients (37.4%) were female.

Preoperatively, mean UDVA was 0.60 ± 0.34 logMAR in eyes with measurable visual acuity (*n* = 157), while inclusion of counting-fingers eyes assigned a conventional value of 1.8 logMAR yielded an overall mean UDVA of 0.70 ± 0.47 logMAR. Mean preoperative CDVA was 0.061 ± 0.101 logMAR. Mean preoperative spherical equivalent was −2.73 ± 2.56 D, and mean refractive cylinder magnitude was 3.29 ± 0.81 D at the spectacle plane (vertex distance 12 mm). Astigmatism was predominantly with-the-rule (94.7%), while against-the-rule and oblique astigmatism accounted for 2.9% and 2.3% of eyes, respectively.

Preoperative corneal data showed mean flat and steep keratometry values of 41.76 ± 1.54 D and 44.96 ± 1.54 D, respectively, corresponding to a mean keratometric astigmatism of 3.20 ± 0.82 D. Mean central corneal thickness was 542 ± 35 μm. In terms of surgical parameters, the mean maximum ablation depth was 71.9 ± 23.5 μm, and the mean residual stromal thickness was 421.9 ± 52.9 μm.

The results are presented in Table 1.

**Table 1:** Demographics, preoperative, and surgical characteristics

Variable	StreamLight™ Trans-PRK (*n* = 171 eyes)
Patient demographics	
Age at surgery (years), mean ± SD	27.87 ± 6.56
Female sex, *n* (%)	64 (37.4%)
Preoperative visual acuity	
UDVA (logMAR), mean ± SD, measurable vision (*n* = 157)	0.60 ± 0.34
UDVA (logMAR, if Counting Fingers is assigned a conventional value of 1.8), mean ± SD (*n* = 171)	0.70 ± 0.47
CDVA (logMAR), mean ± SD (*n* = 171)	0.061 ± 0.101
Preoperative manifest refraction (spectacle plane, vertex distance 12 mm)	
Spherical equivalent (D), mean ± SD	−2.73 ± 2.56
Refractive cylinder magnitude (D), mean ± SD	3.29 ± 0.81
Astigmatism orientation, *n* (%)	
With-the-rule (WTR)	162 (94.7%)
Against-the-rule (ATR)	5 (2.9%)
Oblique	4 (2.3%)
Corneal parameters (corneal plane)	
K1, flat keratometry (D), mean ± SD	41.76 ± 1.54
K2, steep keratometry (D), mean ± SD	44.96 ± 1.54
Keratometric astigmatism (K2 − K1) (D), mean ± SD	3.20 ± 0.82
Central corneal thickness (μm), mean ± SD	542 ± 35
Surgical parameters	
Maximum ablation depth (μm), mean ± SD	71.9 ± 23.5
Residual stromal thickness (μm), mean ± SD	421.9 ± 52.9

Abbreviations: *n* = number of, UDVA = uncorrected distance visual acuity, CDVA = corrected distance visual acuity, logMAR = logarithm of the minimum angle of resolution, SD = standard deviation, D = Diopter, WTR = with-the-rule (astigmatism), ATR = against-the-rule (astigmatism), K1 = flat keratometry, K2 = steep keratometry

### Visual and refractive outcomes

**Fig. 1:**
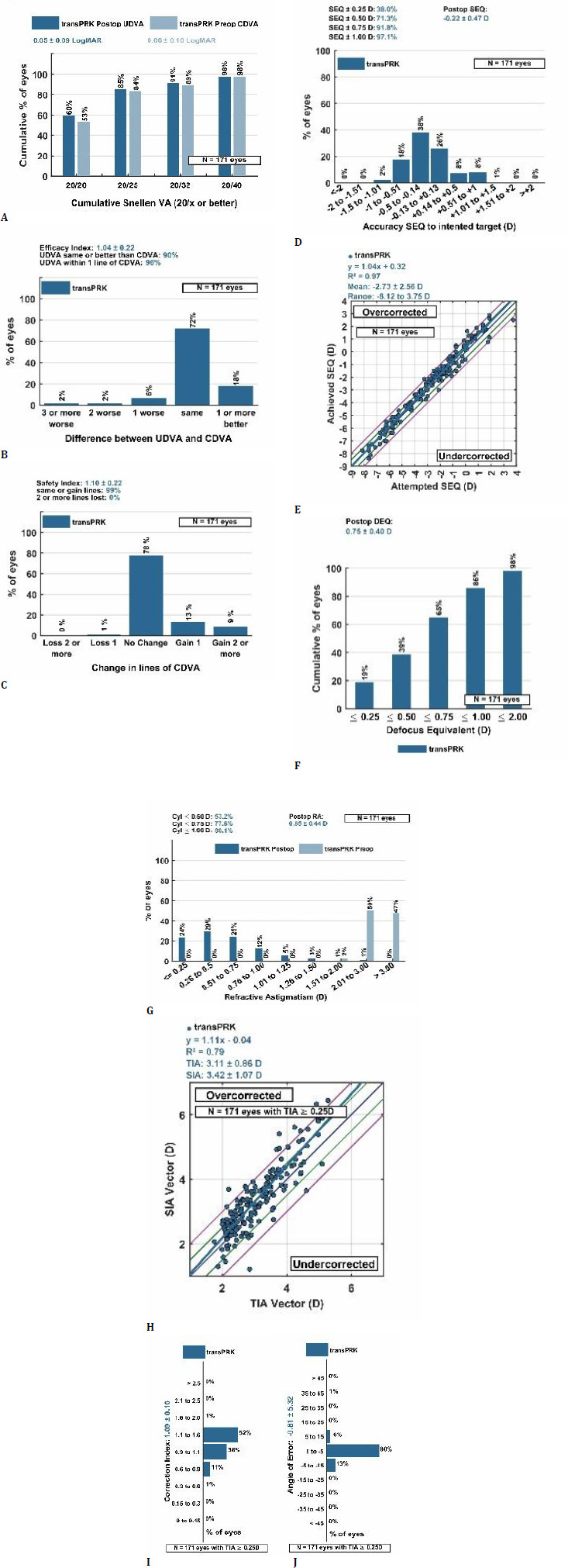
Standard visual and refractive outcomes following StreamLight™ Trans-PRK at 1-month postoperative. A. Cumulative distance visual acuity. B. Distribution of postoperative UDVA relative to corrected distance visual acuity (CDVA). C. Changes in CDVA lines from preoperative to postoperative values. D. Distribution of Spherical Equivalent (SEQ) accuracy relative to the intended target. E. Attempted versus achieved SEQ showing strong linear correlation. F. Defocus Equivalent Histogram. G. Distribution of residual refractive astigmatism. H. Relationship between Target-Induced Astigmatism (TIA) and Surgically-Induced Astigmatism (SIA). I. Distribution of Correction Index (CI). J. Distribution of Angle of Error (AE). Standard graphs were generated from the original data using mEYEstro software, in accordance with current reporting guidelines for refractive surgery outcomes [29]. Abbreviations: UDVA = Uncorrected distance visual acuity, CDVA = Corrected distance visual acuity, N = number of, SEQ = Spherical Equivalent, DEQ = Defocus Equivalent, RA = Refractive astigmatism, D = diopters.

Table 2 summarizes StreamLight™ Trans-PRK results at one month postoperatively. Fig. 1 presents the standard graphs for reporting refractive surgery outcomes (generated with mEYEstro software [29], version 2.5). At the early postoperative 1-month endpoint, mean UDVA improved to 0.05 ± 0.09 logMAR, and mean CDVA was 0.03 ± 0.07 logMAR. The efficacy and safety indices were both above unity, with mean values of 1.04 ± 0.22 and 1.10 ± 0.22, respectively. The cumulative postoperative visual acuity graph showed good uncorrected visual acuity, with most eyes achieving good postoperative UDVA (Fig. 1A). Postoperative UDVA was equal to or better than preoperative CDVA in 90% of eyes and within one Snellen line in 96% of eyes (Fig. 1B), indicating high early refractive efficacy. No eye lost ≥ 2 lines of CDVA, while 9% of eyes gained ≥ 2 lines (Fig. 1C), supporting a favorable safety profile.

**Table 2:** Postoperative visual, refractive, and astigmatic vector outcomes at 1month postoperative (*n* = 171 eyes)

Variable	Value
Visual acuity	
UDVA (logMAR), mean ± SD	0.05 ± 0.09
CDVA (logMAR), mean ± SD	0.03 ± 0.07
Efficacy and safety	
Efficacy index, mean ± SD	1.04 ± 0.22
Safety index, mean ± SD	1.10 ± 0.22
UDVA postop ≥ CDVA preop, %	90%
UDVA postop within 1 line of CDVA preop, %	96%
Loss of ≥ 2 lines of CDVA, %	0%
Gain of ≥ 2 lines of CDVA, %	9%
Refractive outcomes — spherical equivalent (spectacle plane)	
Postoperative SE (D), mean ± SD	−0.22 ± 0.47
SE within ± 0.50 D of target, %	71.3%
SE within ± 1.00 D of target, %	97.1%
Refractive astigmatism	
Postoperative residual cylinder (D), mean ± SD	0.65 ± 0.44
Cylinder ≤ 0.50 D, %	53.2%
Cylinder ≤ 1.00 D, %	90.1%
Postoperative DEQ (D), mean ± SD	0.75 ± 0.40
Astigmatic vector analysis (Alpins method [28], corneal plane measurements, vectors calculated using AstigMETRICS [31])	
TIA (D), mean ± SD	3.11 ± 0.86
SIA (D), mean ± SD	3.42 ± 1.07
Difference vector (DV), mean ± SD	0.65 ± 0.44
Correction index (CI), arithmetic mean ± SD	1.09 ± 0.16
CI distribution	
< 0.9 (undercorrection), %	11%
0.9–1.1 (target), %	36%
> 1.1 (overcorrection), %	53%
Index of success (IOS), mean ± SD	0.21 ± 0.14
Magnitude of error (ME) (D), mean ± SD	0.31 ± 0.50
ME within ± 0.50 D, %	64.9%
ME within ± 1.00 D, %	93.0%
Angle of error (AoE) (°), mean ± SD	−0.81 ± 5.32
AoE within ± 5°, %	80%
AoE within ± 15°, %	99%

Astigmatic vector analysis used measurements at the corneal plane (Alpins’ method [28]), and vectors were calculated with AstigMETRICS software [31] version 1.0. Abbreviations: UDVA = Uncorrected distance visual acuity, CDVA = corrected distance visual acuity, logMAR = logarithm of the minimum angle of resolution, SD = standard deviation, D = Diopter, SE = Spherical Equivalent, DEQ = Defocus Equivalent, TIA = Target-induced astigmatism, SIA = Surgically-induced astigmatism, DV = Difference vector, CI = Correction index, IOS = Index of success, ME = Magnitude of error, AoE = Angle of error

Postoperative spherical equivalent had a slightly myopic mean of −0.22 ± 0.47 D. The attempted versus achieved spherical equivalent scatterplot demonstrated good refractive predictability with a close correlation between intended and achieved correction (Fig. 1E) that yielded a slope of 1.04 (R^2^ = 0.97). Overall, in terms of accuracy, a total of 71.3% of eyes were within ± 0.50 D of the target (emmetropia target), and 97.1% were within ± 1.00 D (Fig. 1D).

Mean postoperative residual refractive cylinder was 0.65 ± 0.44 D, with 53.2% of eyes achieving residual cylinder ≤ 0.50 D and 90.1% achieving ≤ 1.00 D (Fig. 1G). As the metric that accounts for both spherical equivalent and residual cylinder blur, the postoperative defocus equivalent showed a mean value of 0.75 ± 0.40 D (Fig. 1F).

### Astigmatic vector outcomes

Astigmatic vector analysis according to the Alpins method [28] demonstrated a mean target-induced astigmatism (TIA) of 3.11 ± 0.86 D magnitude and a mean surgically induced astigmatism (SIA) of 3.42 ± 1.07 D, with a mean difference vector of 0.65 ± 0.44 D.

The mean correction index (CI) was 1.09 ± 0.16, indicating a mild overall tendency toward overcorrection. Illustrated in the CI histogram (Fig. 1I), a total of 11% of eyes demonstrated undercorrection (CI < 0.9), 36% were within the target range (CI 0.9-1.1), and 53% showed overcorrection (CI > 1.1).

The attempted-versus-achieved astigmatic correction scatterplot showed a strong linear relationship between TIA and SIA magnitudes (y = 1.11, R^2^ = 0.79; Fig. 1H), with a mild tendency toward overcorrection, consistent with the correction index findings (Fig. 1I).

Mean magnitude of error was 0.31 ± 0.50 D, with 64.9% of eyes within ± 0.50 D and 93.0% within ± 1.00 D (Table 2), in line with the mild overall trend toward overcorrection at the early postoperative endpoint.

The low mean index of success (IOS) of 0.21 ± 0.14 (Table 2) indicates a relatively small residual astigmatic error in relation to the intended correction.

The angle-of-error histogram demonstrated high axis-alignment precision (Fig. 1J), with a mean angle of error of −0.81 ± 5.32°. Eighty percent of eyes were within ± 5°, and 99% were within ± 15° of the intended treatment axes (Table 2 and Fig. 1J).

**Fig. 2:**
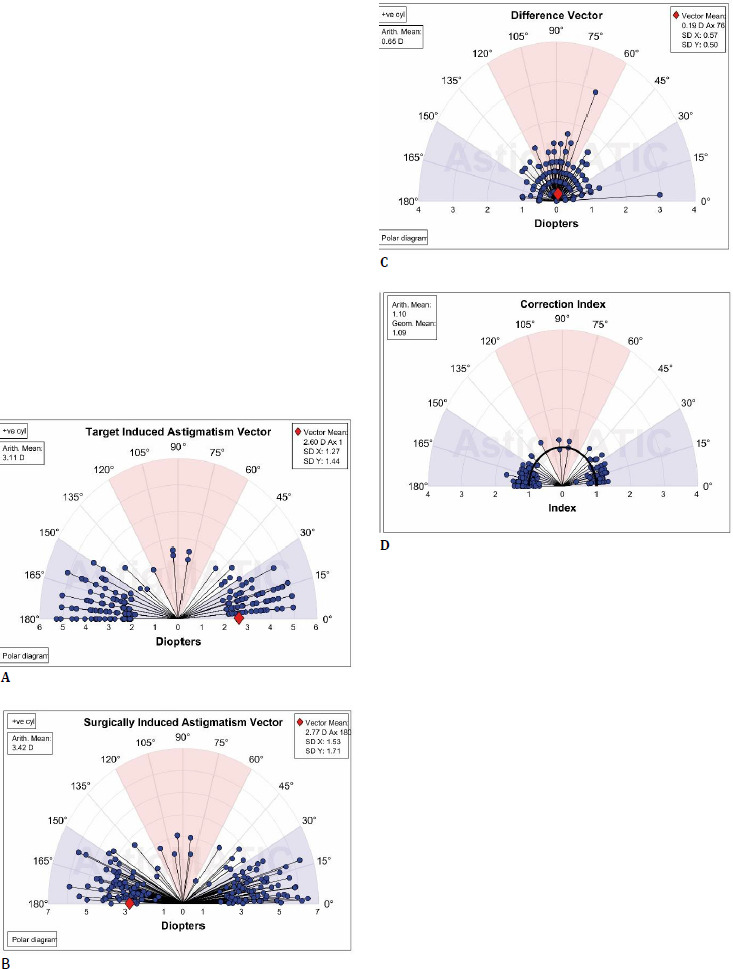
A-D Vectorial astigmatism plots using AstigMATIC [30]. Patients are at 1 month postoperative. The colored regions of the graph represent as follows: with-the-rule astigmatism (WTR) in blue, oblique astigmatism in white, and against-the-rule astigmatism (ATR) in red (orientation convention per the current updated AstigMATIC version 2.0). Each black line represents an individual vector, with the blue point indicating the vectorial arrowhead. The red diamond indicates the position of the mean vector. Abbreviations: D = Diopter, SD = Standard deviation

The TIA and SIA single-angle vector plots showed similar overall vector-orientation distributions, indicating good alignment between the intended and achieved astigmatic correction (Fig. 2). The DV plot showed clustering towards the origin without an evident systematic directional bias (Fig. 2).

Taken together, the visual, refractive, and vector outcomes demonstrated good early postoperative performance of StreamLight™ trans-PRK in eyes with high astigmatism. These findings should, however, be interpreted in the context of the early 1-month follow-up interval, during which refractive stabilization may still be ongoing.

### Stability sub-analysis in eyes with extended follow-up

**Fig. 3:**
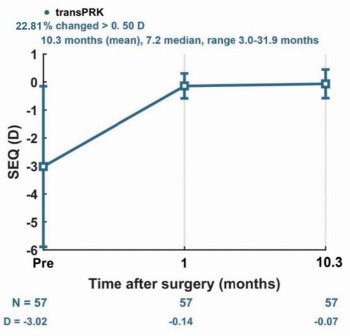
Spherical equivalent stability over time (generated with mEYEstro software, version 2.5.0 [29]). 57 StreamLight Trans-PRK eyes were available with follow-up beyond the 1-month endpoint. The second postoperative timepoint corresponds to the latest available follow-up visit (mean 10.3 months, median 7.2 months, range 3.0-31.9 months), grouped into a single category in the mEYEstro software. Error bars represent ± 1 SD. The annotation reports the proportion of eyes with between-visit (1-month to latest follow-up) SE change > 0.50 D. Abbreviations: SEQ = Spherical Equivalent, N = number of, D = Diopter

Extended follow-up beyond the primary 1-month endpoint was available for 57 StreamLight trans-PRK eyes (33.3% of the cohort), with a mean follow-up duration of 10.31 ± 7.4 months (median 7.2 months; range 3.0-31.9 months). Within-eye outcomes for this subgroup are summarized in Table 3, and the corresponding spherical equivalent stability plot is shown in Fig. 3.

**Table 3:** Secondary descriptive stability sub-analysis in eyes with extended follow-up (*n* = 57)

Variable	Preoperative	1 month	Latest follow-up
UDVA (logMAR), mean ± SD *	0.80 ± 0.57	0.05 ± 0.09	0.03 ± 0.06
CDVA (logMAR), mean ± SD	0.05 ± 0.08	0.02 ± 0.06	0.01 ± 0.03
Spherical equivalent (D), mean ± SD	−3.02 ± 2.87	−0.14 ± 0.45	−0.07 ± 0.51
Refractive cylinder magnitude (D), mean ± SD	3.20 ± 0.84	0.61 ± 0.37	0.61 ± 0.43
Eyes with between-visit SE change > 0.50 D, *n* (%)	—	—	13 (22.8%)

Follow-up duration: mean 10.3 ± 7.4 months, median 7.2 months, range 3.0–31.9 months. *Preoperative UDVA includes counting-fingers eyes assigned a conventional logMAR value of 1.8. Abbreviations: UDVA = Uncorrected distance visual acuity, CDVA = corrected distance visual acuity, logMAR = logarithm of the minimum angle of resolution, SD = standard deviation, D = Diopter, SE = Spherical Equivalent

In the eyes with longer follow-up available, visual acuity was maintained beyond the early 1-month endpoint, with mean UDVA of 0.05 ± 0.09 logMAR at 1 month and 0.03 ± 0.06 logMAR at the latest follow-up, and mean CDVA of 0.02 ± 0.06 and 0.01 ± 0.03 logMAR, respectively.

Within the extended follow-up subgroup, the mean spherical equivalent showed only a small shift, from −0.14 ± 0.45 D at 1 month to −0.07 ± 0.51 D at the latest follow-up (mean within-eye change +0.07 D toward emmetropia), indicating refractive stability at the sub-cohort level. The standard deviations of postoperative SE were similar at the two timepoints (0.45 D and 0.51 D), suggesting that the spread of refractive outcomes did not appreciably widen over time. At the individual-eye level, however, 13 of 57 eyes (22.8%) showed a between-visit SE change of > 0.50 D, indicating that clinically relevant within-eye refractive changes may still occur after the early postoperative period in a subset of eyes.

Mean residual refractive cylinder was essentially unchanged between the 1-month and the latest follow-up visit (0.61 ± 0.37 D and 0.61 ± 0.43 D, respectively), supporting good astigmatic stability after StreamLight™ Trans-PRK in this subgroup. Given the retrospective, real-world nature of the study and the non-uniform follow-up duration, this stability sub-analysis is presented descriptively.

### Postoperative complications

Postoperative complications observed during the 1-month follow-up were infrequent and predominantly mild (Table 4). Corneal haze was the most common postoperative finding, observed in 17 eyes (9.9%); all cases were managed conservatively with topical corticosteroid treatment. Dry eye symptoms were reported in 13 eyes (7.6%) and managed with lubricating eye drops. Other isolated findings included dysphotopsia in 2 eyes (1.2%) and a transient elevation in intraocular pressure in 1 eye (0.6%), which resolved with adjustment of the topical anti-inflammatory regimen.

**Table 4:** Postoperative complications observed during the 1-month follow-up period (*n* = 171 eyes)

Complication	*n* (%)
Corneal haze	17 (9.9%)
Dry eye symptoms	13 (7.6%)
Dysphotopsia	2 (1.2%)
Transiently elevated intraocular pressure	1 (0.6%)

Abbreviations: n = number of

No intraoperative complications, infectious events, visually significant corneal scarring, or other sight-threatening adverse events were observed. No cases of postoperative corneal ectasia were identified at the 1-month primary endpoint, nor in the subgroup of 57 eyes followed for up to 31.9 months (mean 10.3 ± 7.4 months). The limited availability of longer follow-up data should nevertheless be considered when interpreting this finding.

### One-eye-per-patient sensitivity analysis

To assess the robustness of the primary descriptive outcomes to inter-eye correlation in bilateral patients, a sensitivity analysis was performed restricted to one eye per patient (left eye in bilateral cases or the operated eye in unilateral cases), yielding 107 eyes from 107 patients (64 bilateral and 43 unilateral). Mean visual, refractive, and astigmatic vector outcomes in the sensitivity sample were essentially unchanged from the full eye-level analysis (Table 5), with point estimates differing by ≤ 0.03 D for refractive and vector magnitudes, ≤ 0.01 logMAR for visual acuity, and ≤ 0.27° for angle of error. Standard deviations were comparable across the two analyses. The primary descriptive outcomes, therefore, appear robust to potential inter-eye correlations arising from the inclusion of bilateral cases.

**Table 5:** One-eye-per-patient sensitivity analysis: comparison of primary outcomes between the full eye-level analysis and the sensitivity sample

Outcome	Main analysis (*n* = 171 eyes)	Sensitivity (*n* = 107 eyes)
Visual acuity		
UDVA (logMAR), mean ± SD	0.05 ± 0.09	0.06 ± 0.10
CDVA (logMAR), mean ± SD	0.03 ± 0.07	0.03 ± 0.08
Refractive outcomes		
Postoperative SE (D), mean ± SD	−0.22 ± 0.47	−0.22 ± 0.50
Residual cylinder (D), mean ± SD	0.65 ± 0.44	0.67 ± 0.48
Astigmatic vector outcomes (Alpins method, corneal plane)		
TIA (D), mean ± SD	3.11 ± 0.86	3.13 ± 0.86
SIA (D), mean ± SD	3.42 ± 1.07	3.45 ± 1.12
Difference vector (D), mean ± SD	0.65 ± 0.44	0.66 ± 0.48
Correction index, mean ± SD	1.09 ± 0.16	1.09 ± 0.16
Index of success, mean ± SD	0.21 ± 0.14	0.21 ± 0.15
Magnitude of error (D), mean ± SD	0.31 ± 0.50	0.32 ± 0.51
Angle of error (°), mean ± SD	−0.81 ± 5.32	−0.54 ± 5.83

Bilateral patients in the sensitivity sample contributed the left eye only (*n* = 64); unilateral patients contributed their single operated eye (*n* = 43). Vector analysis was performed using AstigMETRICS [31] (version 1.0); refractive and visual analyses were performed in Excel. Abbreviations: *n* = number of, D = Diopters, SE = Spherical Equivalent; SD = Standard Deviation, logMAR, =Logarithm of the Minimum Angle of Resolution; TIA, =Target Induced Astigmatism (Vector), SIA = Surgically Induced Astigmatism (Vector)

## Discussion

This study reports the early refractive, visual, and astigmatic-vector outcomes of single-step StreamLight™ transepithelial photorefractive keratectomy on the WaveLight EX500 platform in 171 eyes selected for a preoperative refractive cylinder of 2.00 dioptres or greater. At one month postoperatively, the cohort achieved a mean uncorrected distance visual acuity of 0.05 logMAR, a mean residual cylinder of 0.65 ± 0.44 D, and mean efficacy and safety indices of 1.04 and 1.10, respectively, with no eye losing two or more lines of corrected distance visual acuity. Corneal-plane vector analysis using the Alpins method [28] yielded a mean correction index of 1.09, an index of success of 0.21, and 99% of eyes within ± 15° of the intended axes. The following sections situate these findings against the published Trans-PRK literature, examine their geometric structure through vector analysis, consider the underlying corneal wound-healing biology, and identify the principal limitations and directions for future work.

The present 1-month outcomes can be situated against published literature that brackets this cohort along the dimensions of platform, technique, and astigmatism magnitude. One comparable series is that of Emerah SH et al., reporting 6-month outcomes of StreamLight™ Trans-PRK on the WaveLight EX500 in a smaller cohort of 30 eyes with a near-identical mean preoperative cylinder of 3.2 D and reporting a residual cylinder ≤ 0.50 D in 70%, ≤ 0.75 D in 100%, and grade 1 haze in 6.6% [32]. The directional convergence with the present series (≤ 0.50 D in 53.2%, ≤ 1.00 D in 90.1%, haze in 9.9%) supports consistency of high-astigmatism StreamLight™ outcomes across centres, with the lower threshold percentages observed plausibly reflecting both the earlier 1-month timepoint at which epithelial remodeling and refractive stabilization remain incomplete (as discussed further below) and the smaller sample of the comparator series. However, no vector analysis was employed in the Emerah study [32].

Another comparator is the larger StreamLight™ series of Gunn and Cox in 205 eyes treated with the same laser and technique [33], but with a substantially lower mean preoperative cylinder of 0.77 D and a mean ablation depth of 52 µm versus 72 µm in the present cohort [33]. Despite the markedly lower astigmatic correction load in their study, the mean efficacy index (1.04) and safety index (1.10) observed in our study slightly exceed their reported values (0.97 and 1.01) [33], supporting the conclusion that StreamLight™ Trans-PRK retains favorable efficacy at the upper end of the astigmatism spectrum.

A third literature reference point is the FS-LASIK versus Trans-PRK comparison in eyes with astigmatism ≥ 2.00 D reported by Reitblat et al. [3,4]. In their series, the single-step Trans-PRK, performed using the Schwind Amaris 500E platform, achieved a mean efficacy index of 0.86 and a mean safety index of 0.89, residual cylinder ≤ 0.50 D in 40.3%, and a correction index of 0.95 ± 0.29 with a mean magnitude of error of −0.13 ± 0.81 D, indicating a tendency toward undercorrection [3,4]. Their corresponding FS-LASIK group performed better on overall efficacy (1.00) and safety (1.03) but showed comparable vector-analysis behavior [3,4]. The present StreamLight™ cohort achieved a residual cylinder ≤ 0.50 D in 53.2%, a correction index of 1.09 ± 0.16, and an index of success of 0.21 ± 0.14, with 99% of eyes within ±15° of the intended axis, values that approach or exceed their Trans-PRK figures and also their FS-LASIK figures (residual cylinder ≤ 0.50 D in 43.0%, IOS 0.32, ± 15° axis precision 95.6%), despite a higher mean preoperative cylinder of 3.29 D versus their 2.72 D. Reitblat et al. acknowledged that their Trans-PRK outcomes were inferior to those of other published transepithelial PRK series using different laser platforms [3,4] and thus the comparison observed here could therefore plausibly be in part attributable to platform-level differences. A formal multi-platform comparison of single-step transepithelial PRK implementations remains beyond the scope of this single-cohort report; the present findings should be interpreted as platform-specific outcomes for the WaveLight® EX500® StreamLight™ protocol rather than as evidence for the technique in general.

Detailed vector-analysis findings, discussed below, complement these overall efficacy and predictability outcomes.

The conventional efficacy and predictability metrics presented above characterize astigmatic outcomes in terms of cumulative refractive shift but provide limited information about the geometric accuracy of the surgically delivered correction. Vector analysis, particularly at the corneal plane, is now considered the methodological standard for evaluating astigmatic correction following corneal refractive surgery and is especially informative in cohorts with high preoperative cylinder, where small angular errors can translate into clinically meaningful residual astigmatism [28,35,3,6]. The present analysis was performed using the mEYEstro [29] and AstigMETRICS [31] software tools, which generate the standard graph reporting set and perform vector calculations in accordance with the Alpins methodology [29,31]. The mean correction index of 1.09 ± 0.16 falls slightly above the unity benchmark established by the meta-analysis of Sabau et al. (1.01, 95% CI 1.01-1.02; 1,924 eyes pooled across 16 studies) [37], possibly reflecting the higher mean target-induced astigmatism (3.11 D) characteristic of cohorts selected for high cylinder. The mean index of success of 0.21 ± 0.14, normalized to the target and therefore directly comparable across studies, is modestly above Sabau’s pooled value of 0.12 [37]. The mean difference vector of 0.65 D is not directly comparable to Sabau’s pooled value of 0.20 D [37] without target-induced astigmatism normalization, since difference vector magnitude scales with the magnitude of the intended correction.

This pattern is consistent with the broader literature in which vector-analysis parameters appear largely independent of laser refractive technique. Comparing 218 eyes treated by Trans-PRK or femtosecond-LASIK in low-to-moderate astigmatism using the Alpins method [28,38], Sun et al. reported no statistically significant difference between techniques in correction index (1.03 *vs*. 1.01; *p* = 0.815), index of success (0.15 *vs*. 0.06; *p* = 0.125), or any other vector parameter [38]. Reitblat et al. reached the same conclusion in eyes with astigmatism ≥ 2.00 D, finding no significant difference between Trans-PRK and FS-LASIK in surgically induced astigmatism, difference vector, correction index, or index of success [3,4].

High axis precision was observed in the present cohort, with a mean angle of error of −0.81 ± 5.32°, with 80% of eyes within ± 5° and 99% within ± 15° of the intended axes, indicating a narrow axis-precision distribution. Reitblat et al. reported 95.7% within ± 15° for their Trans-PRK group and 95.6% for their FS-LASIK group [3,4], while Xi et al. reported 80.9% within ± 10° in low-to-moderate astigmatism eyes treated with Trans-PRK [39].

The interpretation of the present 1-month outcomes requires explicit consideration of the corneal wound-healing biology specific to surface ablation, which differs from that of flap-based procedures in both tempo and the cellular populations involved [40]. Trans-PRK removes the entire epithelial layer over the ablation zone in the same step as the stromal ablation, and the postoperative refractive trajectory therefore reflects the combined contributions of stromal recovery, epithelial regeneration, and longer-term epithelial remodeling [41,42]. Evaluating 52 myopic PRK eyes by anterior-segment optical coherence tomography at 1, 3, and 6 months [42], Sedaghat et al. demonstrated that mean corneal epithelial thickness is significantly reduced in all zones at 1-month relative to the preoperative baseline [42], before progressively thickening to exceed preoperative values centrally and paracentrally by 6 months in a lenticular pattern that mirrors the ablation profile [42]. Importantly, they found a significant correlation between changes in paracentral epithelial thickness and spherical equivalent from the preoperative time point to 6 months postoperatively [42], establishing that epithelial remodeling continues to influence the manifest refraction well beyond the 1-month time point at which the present outcomes were measured [42].

The same temporal pattern has been confirmed specifically for transepithelial PRK by Xu et al., who showed that mean central epithelial thickness decreases during the first postoperative week, returns to approximately baseline at 1 month, and progressively thickens through 6 months [43]. In a comprehensive review of corneal regeneration after Trans-PRK [40], Tomás-Juan summarized the broader pattern: central corneal epithelial thickness reaches the preoperative level at approximately 1-month and continues to increase progressively [40], reaching values up to 21% above baseline by 1 year [40], with epithelial hyperplasia recognized as a contributor to the gradual myopic regression observed in the medium-term postoperative course [40]. The internal stability sub-analysis presented in the “Results” section, comparing 1-month outcomes against an additional follow-up timepoint at a mean of 10.3 months in 57 eyes, showed minimal change in mean spherical equivalent (−0.14 D to −0.07 D) and no change in mean residual cylinder (0.61 D to 0.61 D), supporting that the early outcomes reported here are unlikely to drift substantially in the medium term despite the ongoing epithelial remodeling described above.

The 9.9% incidence of corneal haze observed at 1-month postoperative merits separate consideration within the wound-healing literature. Wilson SE distinguished between two temporally and mechanistically distinct forms of post-PRK haze [44]: an early “common haze” that appears within the first weeks, is approximately proportional to ablation depth, and resolves spontaneously [44], and a later myofibroblast-driven haze that typically peaks at 2 to 3 months postoperatively and is associated with persistent disruption of the epithelial basement membrane [44]. The early timing and uniformly mild presentation of the haze observed in the present cohort might be more consistent with the former mechanism, and the observed response to topical anti-inflammatory treatment suggests a good visual prognosis, with more follow-up data beyond 1 month necessary to ascertain a definitive conclusion. The intraoperative use of mitomycin C targets the latter mechanism described by Wilson SE by inhibiting myofibroblast differentiation in the anterior stroma during the critical post-ablation window [40,44]. The absence of vision-threatening late haze in the stability sub-analysis cohort is consistent with this prophylactic effect.

In our study, Trans-PRK presented a clinically favorable epithelial healing profile, with bandage contact lens removal performed on time. Furthermore, slit-lamp-evaluated reepithelialization showed a consistent appearance across operated eyes, and no eyes in the present cohort lost two or more lines of corrected distance visual acuity at 1 month. Taken together, this data is consistent with the faster epithelial healing reported for single-step Trans-PRK relative to alcohol-assisted PRK by Gaeckle et al. [45], who measured a mean reepithelialization time of 45.8 hours for StreamLight™ Trans-PRK on the WaveLight® EX500® versus 59.1 hours for conventional PRK in a contralateral eye comparison (*p* < 0.0001) [45]. The single-step removal of epithelium and stroma using the same laser source, with continuous patient fixation and uninterrupted tracking, plausibly reduces the cumulative epithelial trauma compared to two-step protocols and could contribute to a smoother early postoperative course. These mechanistic considerations also frame the principal limitation of the present study, namely the early follow-up time point at which outcomes were assessed, which is addressed in greater depth below.

The principal limitation of the present study is the short primary follow-up interval of 1 month, which captures the immediate postoperative outcomes of high-astigmatism StreamLight™ Trans-PRK but does not allow definitive characterization of medium- or long-term refractive stability, late-onset haze, or epithelial-remodeling-driven regression assessment. The internal stability sub-analysis in 57 eyes with a mean follow-up of 10.3 months provides reassurance against substantial drift but is not equivalent to a structured medium-term cohort assessment. Tiskaoglu et al., reporting 36-month outcomes for Trans-PRK and conventional PRK in moderate-to-low myopia and astigmatism, observed continued maintenance of efficacy and safety beyond the early postoperative period [46]; however, long-term data specifically for StreamLight™ Trans-PRK in eyes with preoperative cylinder ≥ 2.00 D falls into a literature gap, and the present cohort would benefit from prospective re-assessment at 12 and 24 months.

Other limitations should be noted. First, the real-world retrospective single-center design carries the methodological constraints inherent to such studies, including selection bias related to surgical indications, surgeons’ preferences regarding surgical decisions, and the absence of randomization. Second, the analysis was restricted to patients treated by the same surgeon with the same laser platform, which strengthens internal consistency but limits the direct generalizability of the findings to other StreamLight™ implementations or other operators. Third, the absence of a contemporaneous comparator arm precludes direct claims of relative performance against alternative refractive techniques.

Future work might address two distinct comparative axes. The first is platform-level variation between single-step transepithelial PRK implementations, which the present findings cannot resolve. The second is technique-level positioning relative to FS-LASIK, SMILE, and toric phakic intraocular lens implantation in the high-astigmatism population, where each technique presents distinct theoretical advantages and limitations, as well as within-platform choice of ablation profile. Prospective comparative studies with structured medium-term follow-up are warranted to clarify the optimal refractive strategy in this challenging population.

## Conclusion

StreamLight™ transepithelial photorefractive keratectomy delivered good early visual, refractive, and astigmatic vector outcomes in this single-center, real-world cohort of eyes with high preoperative refractive astigmatism (≥ 2.00 D), with predictable astigmatic correction, refractive accuracy clustered close to emmetropia, and an acceptable safety profile at the one-month endpoint. Vector analysis demonstrated a close-to-unity correction index of 1.09, a mean residual difference vector magnitude of 0.65 D, and high axial precision, with 99% of eyes within ± 15° of the intended axes at the one-month endpoint. However, the results reflect early postoperative outcomes, and longer-term follow-up is needed to confirm their stability and to extend the surveillance window for late complications such as corneal ectasia or haze. Furthermore, larger comparative studies are warranted to position StreamLight™ Trans-PRK against alternative refractive techniques in this high-astigmatism population.
